# Laparoscopic treatment of median arcuate ligament syndrome without ganglionectomy of the celiac plexus in the hybrid operating room: Report of a case

**DOI:** 10.1016/j.ijscr.2021.105840

**Published:** 2021-03-26

**Authors:** Hiroto Kayashima, Ryosuke Minagawa, Shoichi Inokuchi, Tadashi Koga, Nobutoshi Miura, Kiyoshi Kajiyama

**Affiliations:** a Department of Surgery, Iizuka Hospital, 3-83 Yoshio-machi, Iizuka, Fukuoka, 820-8505, Japan; b Department of Radiology, Iizuka Hospital, 3-83 Yoshio-machi, Iizuka, Fukuoka, 820-8505, Japan

**Keywords:** CA, celiac artery, CPB, celiac plexus block, CT, computed tomography, LGA, left gastric artery, MAL, median arcuate ligament, MALS, median arcuate ligament syndrome, PDA, pancreaticoduodenal artery, SMA, superior mesenteric artery, Median arcuate ligament syndrome, Laparoscopy, Hybrid operating room, Intraoperative angiography, Celiac plexus

## Abstract

**Introduction:**

Median arcuate ligament syndrome (MALS) is a rare condition in which the median arcuate ligament (MAL) causes compression of the celiac artery (CA) and plexus. Although 13–50 % of healthy population exhibit radiologic evidence of the CA compression, the majority remains asymptomatic. With or without symptoms, MALS have a risk of developing collateral circulation that leads to pancreaticoduodenal artery (PDA) aneurysms that have high risk of rupture. The treatment of MALS is the surgical release of the MAL. However, the necessity of ganglionectomy of the celiac plexus is still unclear.

**Presentation of case:**

A 60-year-old man with a ruptured PDA aneurysm caused by MALS was admitted to our hospital for an emergency. After treatment for the ruptured PDA aneurysm by transcatheter arterial coil embolization, he underwent elective laparoscopic MAL release in the hybrid operation room to check blood flow of the CA intraoperatively. The angiography of the CA immediately after MAL release without ganglionectomy of the celiac plexus showed the antegrade blood flow to the proper hepatic artery instead of the retrograde flow via the pancreaticoduodenal arcade. The postoperative course was uneventful and the follow-up computed tomography revealed no residual CA stenosis.

**Discussion:**

Unlike symptomatic MALS, it might be enough to just release the MAL without ganglionectomy of the celiac plexus for asymptomatic MALS, especially that with the treated PDA aneurysm.

**Conclusion:**

Laparoscopic treatment of MALS in hybrid operating room could allow for adequate MAL release without ganglionectomy of the celiac plexus using the intraoperative angiography of the CA.

## Highlights

• The treatment of median arcuate ligament syndrome is the surgical release of the ligament.
• Symptomatic patients need the ligament release with wide excision of the celiac plexus.
• However, the majority of the patients with celiac artery compression remains asymptomatic.
• It might be enough to just release the ligament without ganglionectomy for asymptomatic patients.
• Hybrid operating room could allow for adequate ligament release without ganglionectomy.

## 1 Introduction

Median arcuate ligament syndrome (MALS) is a rare condition characterized by abdominal pain attributed to compression of the celiac artery (CA) and plexus by the median arcuate ligament (MAL), which is a fibrous band formed of crossing the right and left diaphragmatic crura at the level of the CA [1]. MALS has attracted interest in relation to pancreaticoduodenal artery (PDA) aneurysms arising from the collateral vessels between the CA and the superior mesenteric artery (SMA) [2]. Once a PDA aneurysm is diagnosed, it should be treated promptly as ruptured aneurysms are life-threatening and the risk of rupture is not related to the size of the aneurysm [3].

Initial attempts to treat MALS by endovascular angioplasty or stenting are not recommended, because the extrinsic compression of the CA may cause recoil restenosis, dissection, and fracture of the stent [4]. Definitive treatment for MALS involves surgical release of the MAL, and recently a laparoscopic approach has been used by surgeons due to its minimally invasive manner [4,5]. It has been reported that MAL release with ganglionectomy of the celiac plexus is necessary for the result in a decrease of the pain associated with MALS [6]. However, the incidence of symptomatic MALS is 0.4 %, and the majority remains asymptomatic [7]. After all, a standard operation for MAL release has not been established, and the necessity of ganglionectomy of the celiac plexus is still unclear.

We herein report on a case of laparoscopic treatment of MALS in hybrid operating room that could allow for adequate MAL release and avoid excessive resection of the celiac plexus using the angiography of the CA immediately after MAL release without ganglionectomy of the celiac plexus. This work is reported in line with the SCARE 2020 criteria for case report publication [8].

## 2 Case presentation

**Fig. 1:**
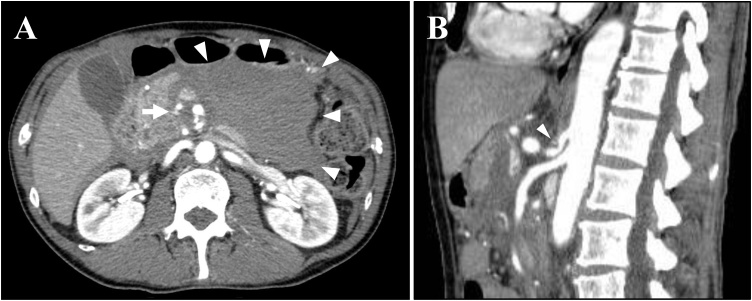
The findings of CT on admission: (A) Axial images showed a massive retroperitoneal hematoma around pancreas (white arrowheads) and a PDA aneurysm on pancreas head (white arrow). (B) Sagittal view of the CT angiography showed extrinsic compression of the root of the CA by the MAL (white arrowhead).

**Fig. 2:**
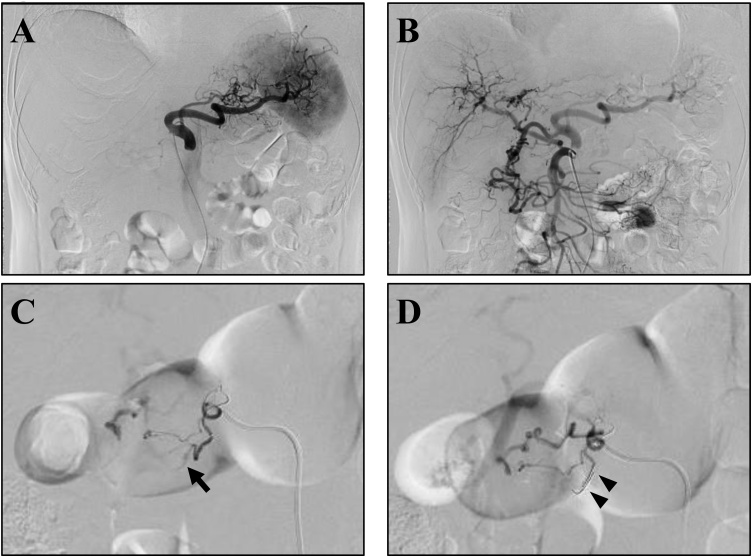
The findings of emergency angiography: (A) The CA angiography showed no antegrade blood flow to the proper hepatic artery. (B) The SMA angiography revealed the retrograde flow via the pancreaticoduodenal arcade in addition to the replaced right hepatic artery. (C, D) The PDA aneurysm (black arrow) was treated by transcatheter arterial coil embolization (black arrowheads).

The patient was a 60-year-old man who was admitted to our hospital for an emergency with severe abdominal pain. He did not take any regular medication. He had smoked a packet of cigarettes a day for 10 years and was a social drinker. He had no allergy and no family medical history. The enhanced contrast abdominal computed tomography (CT) showed a massive retroperitoneal hematoma around pancreas and a PDA aneurysm on pancreas head (Fig. 1A). A sagittal view of the CT angiography revealed extrinsic compression of the root of the CA by the MAL (Fig. 1B). His diagnosis was determined as MALS with a ruptured PDA aneurysm. His vital signs were within normal limits, therefore, we performed endovascular treatment for a ruptured PDA aneurysm at first. The angiography of the CA showed no antegrade blood flow to the proper hepatic artery (Fig. 2A), and that of the SMA revealed the retrograde flow via the pancreaticoduodenal arcade in addition to the replaced right hepatic artery (Fig. 2B). The ruptured PDA aneurysm was treated by transcatheter arterial coil embolization (Fig. 2C, D). 5 days after the treatment, he was discharged with no complication. Although he had no symptom caused by MALS after the treatment for a ruptured PDA aneurysm, there was always a risk of reformation of the PDA aneurysms. Therefore, we planned to perform elective laparoscopic treatment for MALS without ganglionectomy of the celiac plexus in the hybrid operation room to check blood flow of the CA intraoperatively.

**Fig. 3:**
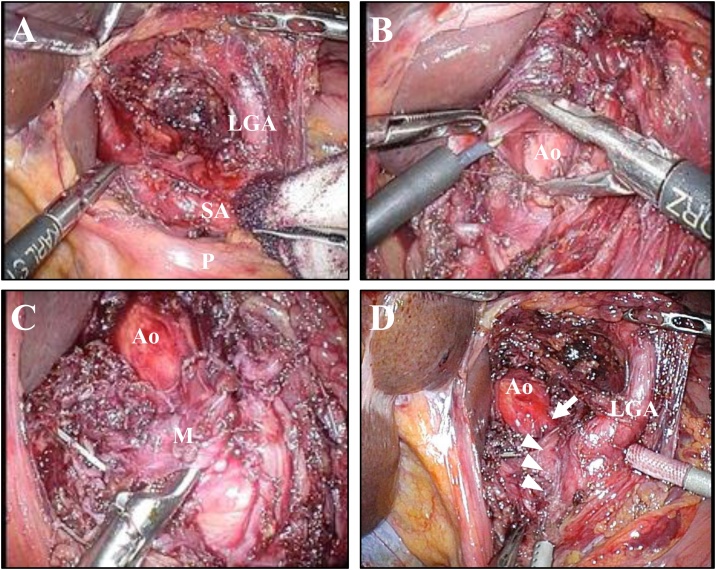
The intraoperative findings: (A) Division of the lesser omentum. (B) Division of the anterior aspect of the aorta. (C) Identification of the MAL. (D) Resection of the MAL and preservation of the celiac plexus. The root of the CA was exposed sufficiently (white arrow) and the celiac plexus was preserved as much as possible (white arrowheads). LGA: left gastric artery; SA: splenic artery, Ao: Aorta; M: MAL; P: pancreas.

**Fig. 4:**
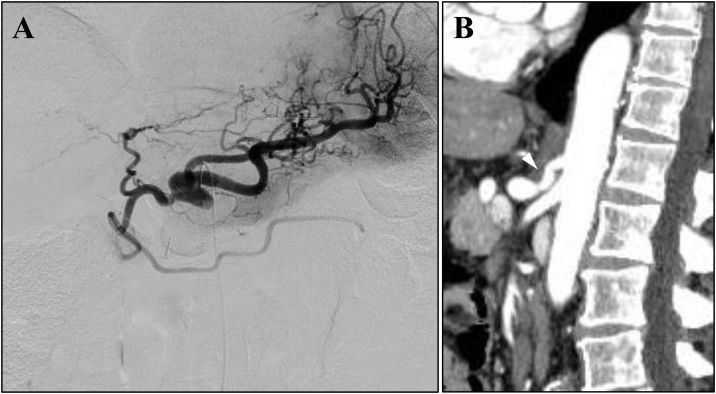
The findings of intraoperative angiography and postoperative follow-up CT: (A) The CA angiography after the MAL release without ganglionectomy of the celiac plexus showed the antegrade blood flow to the proper hepatic artery clearly. (B) The follow-up CT performed 12 months after the surgery revealed no residual CA stenosis (white arrowhead).

The procedure was as follows: The patient was placed in the supine position with a 30 degree both legs opened. An open method was used to insert a 12-mm umbilical camera port, two 5-mm ports in the left upper abdominal, and a 10-mm and a 5-mm port in the right upper abdomen. After liver retraction, a Harmonic Scalpel^®︎^ (ETHICON, Tokyo, Japan) was used to divide the avascular region of the lesser omentum, and the left gastric artery (LGA) was identified on the suprapancreatic surface (Fig. 3A). While retracting the LGA, the anterior aspect of the aorta was divided adequately (Fig. 3B). The MAL was identified as a musculoaponeurotic band crossing over the anterior surface of the CA (Fig. 3C). The MAL was resected and the root of the CA was exposed sufficiently, and the celiac plexus was preserved as much as possible (Fig. 3D). Immediately after the MAL release without ganglionectomy of the celiac plexus, the angiography of the CA was performed to check blood flow. Unlike previous angiography, the antegrade blood flow to the proper hepatic artery was clearly depicted (Fig. 4A). The surgical operator had 18 years’ operative experience of general surgery and the duration of surgery was 183 min.

His postoperative course was uneventful and he was discharged on postoperative day 5. The follow-up CT performed 12 months after the surgery revealed no residual CA stenosis (Fig. 4B). He was satisfied with his current condition and had no complaint about our treatment.

## 3 Discussion

The CA compression at its origin by diaphragmatic crura was observed by Lipshutz in 1917 [9]. The first clinical case of MALS was described in 1963 as a syndrome attributed to compression of the CA by the MAL [10]. Dunbar et al. reported a series of 15 cases, which presented with postprandial abdominal pain and weight loss, and had angiographic evidence of the CA stenosis. Twelve out of the 15 patients had resolution of symptoms after release of the CA compression by MAL sectioning [1].

Various pathophysiological mechanisms have been suggested to explain the symptoms of MALS but none has been proven. The most accepted theory is that increased blood demand caused by compression of the CA leads to mesenteric ischemia, and another theory is that midgut ischemia causes pain through steal syndrome, wherein blood from the SMA area is diverted through the collateral circulation to compensate for inefficient blood flow from the CA. A third theory is that pain in MALS is caused by a neurogenic dysfunction resulting either directly from compression of the celiac plexus or indirectly from splanchnic vasoconstriction [5,11]. Based on the third theory, complete celiac trunk decompression is recommended and only simple MAL release is not enough, additional neurolysis and wide excision of the celiac plexus is necessary. These procedures have been reported to not only better inhibit reformation of a compression but also the result in a decrease of the pain associated with MALS [6]. However, the incidence of symptomatic MALS is only 0.4 % even though 13–50 % of healthy population exhibit radiologic evidence of the CA compression, and the majority remains asymptomatic [7]. The necessity of ganglionectomy of the celiac plexus for these asymptomatic MALS patients is still unclear.

The celiac plexus, also known as the solar plexus, is the largest plexus of the sympathetic nervous system. It relays sensory innervation of the gastrointestinal tract from stomach to splenic flexure. In animal experimental study about the pathogenesis of acute cholecystitis, ganglionectomy of the celiac plexus alone did not induce acute cholecystitis, but it played an important role in the progression of the inflammatory process in ischemia-reperfusion injury [12]. In another animal experiment, it was reported that the celiac plexus regulated hepatic glucose metabolism and was a critical component in the maintenance of blood glucose homeostasis [13]. Based on these reports, surgical indication of ganglionectomy of the celiac plexus in MALS patients should be considered carefully. It was reported that pain relief after celiac plexus block (CPB) had been found to be a predictor of symptomatic improvement after corrective surgery [14]. Some authors reported that CPB was useful to confirm candidacy for corrective surgery of MALS [15,16]. In these symptomatic MALS patients, MAL release with ganglionectomy of the celiac plexus might be the best treatment.

With or without symptoms, MALS has attracted interest in relation to PDA aneurysms. Mano et al. have described the hemodynamic mechanisms of aneurysmal formation in the collateral vessels between the CA and the SMA, that is, in the presence of compression in the CA, the shear stress on the collateral vessel walls increases remarkably [2]. Once a PDA aneurysm is diagnosed, it should be treated promptly as ruptured aneurysm are life-threatening and the risk of rupture is not related to the size of the aneurysm [3]. However, the necessity of additional treatment for the stenosed CA remains unclear. Some authors have reported that the CA reconstruction might not be necessary given that no patients have developed organ ischemia or relapse of the aneurysm after coil embolization alone. However, the follow-up periods in these reports were not long enough to draw a firm conclusion, as was acknowledged by the authors [17,18]. On the other hand, there are some authors who emphasize the importance of revascularization or decompression of the CA to decrease the stress on the collateral vessel walls between the CA and the SMA and to prevent the formation of new aneurysms [19,20]. Considering these reports, it might be enough to just release the MAL without ganglionectomy of the celiac plexus for additional surgery of the MALS patients with the treated PDA aneurysm.

Our patient had no symptom caused by MALS itself, however, there was always a risk of reformation of the PDA aneurysms. Therefore, we planned to perform elective laparoscopic treatment for MALS without ganglionectomy of the celiac plexus. In this surgery, one of the most important points was to obtain the sufficient antegrade blood flow from the CA to the proper hepatic artery. Using the hybrid operating room, we could check direct blood flow of the CA intraoperatively and allow for adequate MAL release and avoid excessive resection of the celiac plexus.

## 4 Conclusion

Symptomatic MALS patients need MAL release with wide excision of the celiac plexus for complete celiac trunk decompression to decrease the pain associated with MALS. However, it might be enough to just release the MAL without ganglionectomy of the celiac plexus for asymptomatic MALS patients, especially that with the treated PDA aneurysm. Laparoscopic treatment of MALS in hybrid operating room could allow for adequate MAL release without ganglionectomy of the celiac plexus using the intraoperative angiography of the CA.

## Declaration of Competing Interest

The authors declare that they have no competing interests.

## Sources of funding

The authors declare that they have no sponsors to disclose concerning the article.

## Ethical approval

None.

## Consent

Written informed consent was obtained from the patient for publication of this case report and accompanying images. A copy of the written consent is available for review by the Editor-in-Chief of this journal on request.

## Author contribution

HK wrote the manuscript. HK and RM performed the surgery and treatment. NM performed the transcatheter arterial coil embolization and intraoperative angiography. SI and TK discussed the manuscript. RM and KK revised the manuscript. All authors read and approved the final manuscript.

## Regisstration of research studies

Not applicable.

## Guarantor

Hiroto Kayashima.

## Provenance and peer review

Not commissioned, externally peer-reviewed.
